# Strategies to sustain a quality improvement initiative in neonatal resuscitation

**DOI:** 10.4102/phcfm.v8i2.958

**Published:** 2016-04-22

**Authors:** Carlien van Heerden, Carin Maree, Elsie S. Janse van Rensburg

**Affiliations:** 1 Department of Nursing Science, University of Pretoria, South Africa; 2 Department of Health Studies, UNISA, South Africa

## Abstract

**Background:**

Many neonatal deaths can be prevented globally through effective resuscitation. South Africa (SA) committed towards attaining the Millennium Development Goal 4 (MDG4) set by the World Health Organization (WHO). However, SA’s district hospitals have the highest early neonatal mortality rates. Modifiable and avoidable causes associated with patient-related, administrative and health care provider factors contribute to neonatal mortality. A quality improvement initiative in neonatal resuscitation could contribute towards decreasing neonatal mortality, thereby contributing towards the attainment of the MDG4.

**Aim:**

The aim of this study was, (1) to explore and describe the existing situation regarding neonatal resuscitation in a district hospital, (2) to develop strategies to sustain a neonatal resuscitation quality improvement initiative and (3) to decrease neonatal mortality. Changes that occurred and the sustainability of strategies were evaluated.

**Setting:**

A maternity section of a district hospital in South Africa.

**Methods:**

The National Health Service (NHS) Sustainability Model formed the theoretical framework for the study. The Problem Resolving Action Research model was applied and the study was conducted in three cycles. Purposive sampling was used for the quantitative and qualitative aspects of data collection. Data was analysed accordingly.

**Results:**

The findings indicated that the strategies formulated and implemented to address factors related to neonatal resuscitation (training, equipment and stock, staff shortages, staff attitude, neonatal transport and protocols) had probable sustainability and contributed towards a reduction in neonatal mortality in the setting.

**Conclusion:**

These strategies had the probability of sustainability and could potentially improve neonatal outcomes and reduce neonatal mortality to contribute toward South Africa’s’ drive to attain the MDG4.

## Introduction

Neonatal mortality is a worldwide problem and is identified as a priority focus of the World Health Organization (WHO). The Millennium Development Goals 4 (MDG4) set by the WHO aim at reducing the under five years’ old child mortality rate by two-thirds between 1990 and 2015, of which the neonatal mortality (number of deaths within 28 days per 1000 live births) forms a crucial part.^1,2^

The neonatal mortality rate (NMR) forms an important part of the under-five mortality rates. Neonatal deaths refer to the number of infants who died during the first month of life; this period is considered the time of highest risk for child death.^1^ Neonatal deaths can be subdivided into early and late neonatal deaths with early neonatal deaths occurring from birth to seven days and late neonatal deaths being from seven to 28 days.^3^

Worldwide, a decrease in neonatal mortality lagged behind when compared to the under five years’ old mortality rates in 2012, indicating the global newborn mortality rate decreased only by 37% from 33/1000 live births to 21/1000 live births, representing 44% of the total under-five mortality rate.^4^ The current reality is that 2.9 million neonatal deaths still occur worldwide. The percentage of deaths occurring in the neonatal period increased to 44% as reflected in the latest available statistics.^4,5^ However, this percentage does not include the 2.6 million stillbirths, of which 1.2 million (45%) occur during labour. Furthermore, of these deaths 99% occur in low- and middle-income countries.^6,7^

Data from the Saving Babies Report (2003–2005) indicate the neonatal mortality in South Africa (SA) contributed to approximately 30% of the under-five mortality rate in the early 2000s.^1^ The neonatal deaths in SA had, in fact, increased since 2011 as they accounted for approximately 40% of the under-five mortality rate in the country in 2013.^8^ Data from Statistics SA (StatsSA) indicated the NMR for SA was 14/1000 live births in 2008, and that there was no change in the NMR from 2000–2008.^8,9^ The Rapid Mortality Surveillance Report 2011 indicates the NMR for SA was 14/1000 live births in 2011.^10^ In the Saving Babies Report of 2010–2011 (which reports on the Perinatal Problem Identification Programme (PPIP) data) the NMR is indicated as 21/1000 live births.^8^

In addition to the causes of neonatal deaths related to immaturity and health problems, there are also avoidable and modifiable health care problems that contribute to these high mortality rates.^1,11,12,13^ Avoidable and modifiable health care problems include health care provider and administrative problems that lead to inadequate care to prevent morbidity and mortality, or to health problems not being appropriately managed.

The early NMRs are the highest in SA’s district hospitals.^14^ Despite clear guidelines and recommendations set by the Saving Babies Report,^3,13^ and recurring in consecutive reports,^3,13,15^ neonatal mortality remains a challenge for SA. The abovementioned problems were also applicable to the specific district hospital where this research study was conducted.

Competent neonatal resuscitation is a critical intervention for survival of infants in need of respiratory or cardiac support.^16^ Inadequate neonatal resuscitation contributes to neonatal morbidity and mortality in South African district hospitals.^3,13^ Factors contributing to inadequate neonatal resuscitation are the lack of staff, inadequately trained staff, limited equipment for resuscitation and the lack of well-equipped transport facilities.^3,13,16^ Effective resuscitation is a competency that needs to be learned and practised in an appropriate environment. The high neonatal mortality in district hospitals and the lack of improvement over the past decade indicate a complex and multifaceted lack of sustained quality care that impacts negatively on neonates.^3,17^ A premise of this study was that placing the lives of neonates at risk is unacceptable and calls for a quality improvement initiative with clear strategies to sustain quality resuscitation of neonates in district hospitals.

To improve and sustain the practice of neonatal resuscitation in the long-term implies addressing the process, staff and organisational issues. Quality improvement initiatives have a 70% chance to fail because of the lack of sustainability.^18^ For quality improvement initiatives to be sustainable, a holistic approach should be followed, and the focus should be on the factors regarding process, staff and organisation that are captured by the National Health Service (NHS) Sustainability Model.^18^

The NHS Sustainability Model focuses on 10 factors relating to process, staff and organisation in sustaining change. The Sustainability Guide of this model provides practical advice on how to increase the likelihood of sustainability for a quality improvement initiative, and the Master Score System, which forms part of the Sustainability Model, is an evaluation instrument which determines whether a quality improvement initiative is sustainable.^18^ The NHS Sustainability Model and Guide formed the theoretical framework of this study.

A successful long-term initiative to reduce neonatal mortality and morbidity in the particular district hospital needed to address issues related to health care provider and administrative problems. The researcher, in collaboration with the stakeholders, embarked on a quality improvement process to initiate and sustain change in neonatal resuscitation using action research. This quality improvement initiative focused on neonatal resuscitation and factors related to the process, staff and organisation of a district hospital that could have an influence on effective neonatal resuscitation. The sustainability of these strategies could improve neonatal mortality, thereby contributing towards the attainment of the MDG4, which SA has not yet achieved.

### Aim of this study

The overall aim of this study was to explore and describe the existing situation in the specific district hospital in order to develop strategies to sustain the quality improvement initiative implemented in neonatal resuscitation for decreasing neonatal mortality (which is the focus of MDG4). In addition, to determine what changes occurred as a result of these strategies and if the changes were sustainable.

The objectives of this study were the following:

- To explore and describe the existing practices of neonatal resuscitation and the factors influencing neonatal resuscitation – (CYCLE 1).
- To develop and implement strategies to sustain the quality improvement initiative in neonatal resuscitation – (CYCLE 2).
- To determine what changes occurred as a result of the neonatal resuscitation strategies that were implemented – (CYCLE 3).
- To determine the sustainability of these strategies – (CYCLE 3).

The strategies were expected to bring about positive sustainable change in practice. Consequently, the improved change could become the norm of practice. Additionally, it could serve as a benchmark for the implementation of future quality improvement initiatives on other aspects of care in this particular hospital, as well as for improved neonatal resuscitation in other health care settings (within a similar context) in SA and, in addition, contributing towards the attainment of SA’s MDG4.

## Research methods and design

### Study design

The action research design of choice for this study was the Problem Resolving Action Research (PRAR) model.^19^ The PRAR model is a spiralling process of planning, acting, observing and reflecting. The model is spiralling upward in direction to indicate the observation that action research is a continuous improvement approach.^19^ Using the PRAR model in this study meant the research process took place in cycles. In each cycle of the research process there were four steps: planning, action, observation, and reflection. Consequently, research and problem-solving occurred simultaneously which contributed towards addressing practical concerns where a problem had been identified.^19^

CYCLE 1 focused on addressing the objective to explore and describe the existing practices of neonatal resuscitation, and the factors influencing neonatal resuscitation. CYCLE 2 focused on the objective to develop and implement strategies to sustain the quality improvement initiative in neonatal resuscitation. CYCLE 3 focused on the objective to determine what changes occurred as a result of the strategies implemented and their sustainability.

### Setting

The setting was a maternity section of a district hospital (level 2) situated in a rural area in the Gauteng province in SA. Patients are referred from 32 surrounding clinics to this district hospital for care. The maternity section comprises a 60 bed postnatal ward, a labour ward with 10 labour rooms and a 35 bed neonatal intensive care unit (NICU) that includes a 12 bed kangaroo mother care unit (KMCU) and four intensive care unit (ICU) beds. Babies are mainly delivered in the labour ward and mostly by midwives. Facilities for caesarean sections are also available. After delivery, the healthy newborns and their mothers are transferred to the postnatal ward. Sick or premature newborns are transferred to the NICU for specialised treatment. If a newborn baby needs resuscitation at birth, the midwife or available doctor resuscitates the baby before transferring the baby to the NICU.

### Study population and sampling

As the quality improvement initiative was part of an action research study, the population in all three cycles was the entire group of individuals caring for neonates in the maternity section in the selected district hospital.

The criteria for inclusion in this study were all health care providers (doctors and nurses) delivering and caring for neonates in the maternity section of the district hospital irrespective of their years of working experience or post-basic type of qualification. The inclusion criteria were the same for both the quantitative and qualitative data. The individuals, though, had a choice if they wanted to complete the questionnaires and to participate voluntarily in the focus groups interviews or nominal group technique discussion (NGT).

### Discussion of cycles

**FIGURE 1:**
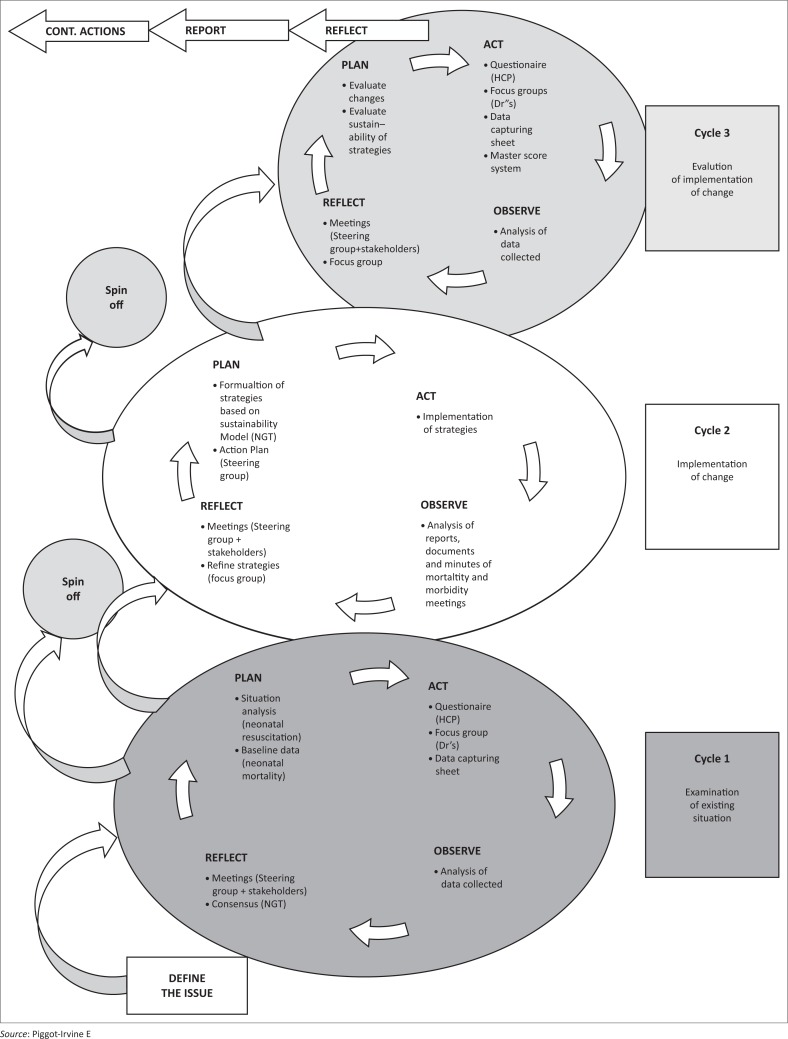
Problem resolving action research cycles.

The study was conducted in three cycles as illustrated in Figure 1. Before commencing with the study, a steering group was established.

### CYCLE 1: Situation analysis

The focus of CYCLE 1 was on the objective to explore and describe the existing practices of neonatal resuscitation and the factors influencing neonatal resuscitation.

The first step, *plan*, for CYCLE 1 included a situational analysis and the gathering of baseline data. The situational analysis focused on the practice of neonatal resuscitation and other influencing factors related to process, staff and organization and neonatal mortality as the indicator of quality of care. The second step, *act*, dealt with data gathering. The data gathering tools included a questionnaire,^20^ focus group^21^ and data capturing sheets. The sample for the questionnaire was all categories of nurses (*n* = 69) working in the maternity section, and the sample for the focus group included the doctors (*n* = 8) (including specialists). The unit of analysis for the data capturing sheets were documents and records pertaining to statistics related to neonatal mortality, number of births etc. The data capturing sheets provided baseline data for neonatal mortality. During the third step (*Observe*) data from previous steps were analysed. The quantitative data were analysed by making use of descriptive statistics^22^ and inferential statistics to make inferences about the population and cross-tabulations.^23^ The qualitative data were analysed by open-coding using Tesch’s method of open-coding.^24^ Lastly, step four followed, *reflection,* where consensus was reached by means of a nominal group technique (NGT) discussion^25^ with the steering group and the doctors and nursing staff from the maternity section (*n* = 10). Reflective meetings were also held with the steering group.

### CYCLE 2: Implementation of strategies

The focus of CYCLE 2 (Figure 1) was to develop and implement strategies to sustain the quality improvement initiative in neonatal resuscitation. The first step, *plan*, was to develop and formulate strategies based on the findings from CYCLE 1, consensus reached during the NGT and a literature control. In the second step, *act*, strategies were implemented. During the third step, *observe*, documents and reports were supposed to be analysed, but the documentation regarding the implementation of the strategies was insufficient. The researcher, therefore, relied on the minutes of meetings held by the steering group and a feedback meeting held with them regarding the implementation of strategies. The fourth step, *reflect*, included a reflective meeting that was held with the steering group members regarding the implementation and refinement of strategies.

### CYCLE 3: Evaluation of strategies and determining the sustainability of the strategies

The focus of CYCLE 3 (Figure 1) was to determine the changes as a result of the neonatal resuscitation strategies that were implemented and to determine the sustainability of these strategies. During the first step, *plan*, the changes and sustainability of the strategies were evaluated. During the second step, *act*, various data gathering tools were used to collect data including a questionnaire,^20^ data capturing sheets to provide data on neonatal mortality, etc. and Master Score System.^15^ The sample for the questionnaire was the same as in CYCLE 1 and included all the categories of nurses working in the maternity section of the district hospital (*n* = 71). The Master Score System served as a tool to evaluate the sustainability of the strategies^18^ that were implemented and was completed by the matron, operational managers of the maternity section and the researcher. The doctors were included in the sample for the focus group that was held in step four, *reflection*. During the third step, *observe*, the data from the previous steps of the cycle were analysed. The quantitative data were analysed as descriptive and inferential statistics as well as cross-tabulations to classify responses and compare summary data output on the basis of variables.^23^ The qualitative data were analysed through open-coding. Lastly, step four followed, *reflection,* through conducting a focus group (*n* = 10) with the steering group, doctors and professional nurses.^21^ This focus group served the dual purpose of reflection and evaluation of strategies. Reflective meetings were also held with the steering group.

### Ethical considerations

In this study, the fundamental ethical principles underling the protection of human beings when conducting nursing research involving humans were adhered to. These included the three basic ethical principles guiding researchers, namely respect for persons, justice and beneficence.^23^

Informed consent documents included adequate information with regard to participation and stated clearly that participants were free to choose and could participate voluntarily or decline participation. Approval from various ethical committees (219/2012), Gauteng Department of Health and the district hospital was obtained to conduct this study. The benefits outweighed the risks; there was no risk of exploitation of the study participants. The researcher took the culture and the contemporary health care politics of the context into consideration.

## Results

### CYCLE 1: Situation analysis

The aim was to examine the existing situation regarding neonatal resuscitation prior to the implementation of the quality improvement initiative. Data from the questionnaire, focus group and data capturing sheet were analysed. The findings regarding the existing situation consisted of recurrent themes of challenges that are experienced on a daily basis in the maternity section. They included training, the lack of staff resources and expertise, lack of equipment and stock, aspects influencing staff attitude and challenges with regards to transport and aspects related to the culture of the organisation.

The need for training was identified as important. Factors such as shortage of staff and lack of financial resources prevented staff from attending training opportunities. Training was inconsistent and there were respondents (nursing staff) that had never had the opportunity to attend neonatal resuscitation training, and 89.2% (*n* = 37) had never practised neonatal resuscitation on a mannequin. A need was therefore identified for an in-house training programme specifically aimed at neonatal resuscitation.

Shortages of staff were a recurrent theme and 68.4% (*n* = 38) of respondents (nursing staff) indicated that there were not enough staff to render quality care and quality neonatal resuscitation. Participants from the focus groups indicated that they experienced a lack of staff especially during night duty, and the nurse to patient ratio was very low. At night, the nurse to patient ratio in the labour ward is approximately 1:4, in postnatal ward 1:16 and in the NICU 1:11. There was also a need for senior and clinical leaders with expertise, specifically with regards to neonatal care. Shortages of staff also lead to ethical issues, causing staff to make difficult decisions such as choosing between a mother and her baby, and in general had a negative impact on staff attitude.

Staff attitude (feelings and behaviour) was also identified as a challenge. Staff felt overworked and demoralised because of the challenges they experienced. There was a lack of self-confidence with regards to neonatal resuscitation as seen in the following quotes:

‘… the nursing staff doesn’t have confidence in themselves’ (Participant 1, female, professional nurse); ‘… low level of confidence because they are scared of like something happens then managers will say why did you do something …’ (Participant 5, female, senior professional nurse).

This had a negative influence on staff involvement and willingness to take accountability and responsibility and led to staff avoiding participation in neonatal resuscitation. Other challenges were the lack of a clear vision and aim regarding neonatal resuscitation, and a lack of communication between staff and the management. Challenges staff experienced in their work environment that were not discussed caused staff to feel despondent. Therefore, a need for improvement in communication was identified in order to address challenges and enhance quality care.

There were recurrent themes of lack of essential emergency equipment and stock needed for neonatal resuscitation. Meetings were held to address these challenges, but the procurement process was very slow. The maintenance of equipment was inadequate, for example equipment would be available but was not serviced and maintained and was therefore not operational.

Delay of transport and lack of equipped ambulances with competent emergency services (EMS) staff was a further challenge that contributed towards neonatal deaths. Communication regarding the transport process was also ineffective.

The culture in the hospital with regards to practices and protocols was not always conducive, and protocols were inconsistent and unavailable in some of the units.

In order to reach consensus, generate ideas and prioritise the challenges that had to be addressed, a NGT was held with the steering group, nurses and doctors. These findings formed the basis of CYCLE 2.

### CYCLE 2: Implementation of strategies

The aim was to formulate and implement strategies to sustain a quality improvement initiative in neonatal resuscitation. These strategies were based on the findings of CYCLE 1, consensus reached during the NGT and a literature control. The Sustainability Model served as a theoretical framework.^18^ The strategies addressed the practice of neonatal resuscitation, process, staff and organisation.

Training in neonatal resuscitation was identified as the highest priority, followed by equipment and stock, attitude, shortage of staff, transport and protocols. Box 1 serves as a summary of the strategies for implementation.

### CYCLE 3: Evaluation of strategies and determining their sustainability

The focus was to evaluate the changes that occurred in the maternity section as a result of the implemented strategies. The same questionnaire and data capturing sheets were used as in CYCLE 1. To assess the sustainability of these strategies, the NHS Master Score System was applied.^18^

After the implementation of the strategies (which included neonatal resuscitation^26,27,28^ training opportunities and practise on a mannequin) the percentage of respondents who had never had the opportunity to practise neonatal resuscitation on a mannequin decreased from 89.2% to 15.4%, whilst those who had obtained a monthly opportunity increased from 0% to 56.3%. All staff members had the opportunity to attend training, however communication regarding training and practice opportunities were not optimal and could have been contributing towards the lack of attendance by some of the staff. Staff members who did attend the neonatal resuscitation training and practice opportunities indicated that they were positive experiences that increased their confidence in practising neonatal resuscitation. There was also an increase in staff perceiving themselves as feeling competent regarding neonatal resuscitation. From the findings in CYCLE 3 it could be concluded that 69.7% (*n* = 39) felt confident in comparison to the 35.9% (*n* = 39) in CYCLE 1. After the training, there were also improvements regarding the critical aspects of neonatal resuscitation. Nurses who perceived themselves to be knowledgeable regarding the new trends and guidelines in neonatal resuscitation also felt competent, especially in the NICU. The results showed improvements in the nurses’ knowledge regarding neonatal resuscitation. Competent neonatal resuscitation would contribute towards a decrease in neonatal mortality and would, therefore, contribute towards the MDG4.

**TABLE 1:** Strategies for implementation.

Strategies	Implementation
Strategy to address training	Creating neonatal resuscitation training opportunities
	Placement and orientation of staff
	Enforcing and support for training in neonatal resuscitation
Strategy to address equipment and stock	Needs assessment regarding equipment and procurement of equipment
	In-service training of staff on the use of equipment
	Maintenance plan for servicing of equipment
	Stock control
Strategy to address staff attitude	Task team to address staff attitude
	Staff support (communication, training and support systems)
	Professional conduct
Strategy to address shortage of staff	Ensure optimal functioning of available staff
	Retention strategy for staff that want to resign
	Budget for recruitment of staff and overtime
	Recruitment of new staff
Strategy to address transport for transfer of critically ill neonates	Task team (hospital management and EMS staff) to address concerns regarding transportation
	Availability of equipped transport and competent EMS staff
	Communication regarding transport
Strategy to address protocols	Task team to address protocols regarding neonatal resuscitation
	Awareness of protocols regarding neonatal resuscitation

*Source:* Authors own work

During the quality improvement initiative the maternity section received some equipment such as radiant warmers, mobile suction machines, Neopuffs, ambubags, a mobile incubator and a blood gas machine. Staff received in-service training on the use of some of this equipment. This equipment can potentially contribute towards the prevention of neonatal resuscitation and improve neonatal outcomes and neonatal mortality, which would contribute towards the MDG4. Each of the units received a resuscitation toolbox that contains the necessary emergency equipment and stock needed for neonatal resuscitation. Staff members perceived these boxes as positive as they assist with organisation during resuscitation. There is still a need for some lifesaving equipment such as laryngoscope blades and McGill’s forceps, as well as at least three radiant warmers for the NICU. Positive changes were made regarding equipment control through equipment control books in all three of the units. However, the maintenance of equipment was not fully addressed because of limited financial resources. The management and control of equipment contributed to functionality of the maternity unit. There seemed to be improvement in stock control through the recruitment of knowledgeable staff in the supply chain management of the hospital.

Staff attitude was influenced by many factors related to work environment and work conditions; staff attitude also influenced staff involvement and quality care. The researcher’s reflection was that staff attitude is central to the success of a quality improvement initiative and that something to improve or change the strategies had to be implemented. It was the reflection of the steering group that there was an overall improvement regarding staff attitude in the maternity section. Staff attitude was positively influenced by the training, staff involvement in neonatal resuscitation and improved communication regarding neonatal resuscitation together with the challenges experienced. With the acquisition of the knowledge and skills staff members indicated that they felt more confident and competent in performing neonatal resuscitation. Findings indicated that there was a positive change regarding collaboration and teamwork between the team members and units. Staff members felt supported by management regarding the challenges identified, provided solutions were within their control. Factors that had a negative influence on staff attitude were the lack of certain lifesaving equipment and staff resources. These factors contributed to staff feeling despondent and overworked. Staff members also have a need for emotional support in the form of mortality and morbidity meetings and debriefing sessions after failed resuscitations.

Staff shortages remained a problem in the maternity section, as there had not been significant improvement regarding the recruitment of staff. The number of staff in 2014 compared to 2013 was the same for the labour and postnatal wards, and the NICU recruited one professional nurse. The nurse to patient ratio was still the same as for CYCLE 1. The ratios were determined based on the average number of patients daily. There was a high turnover of staff, leaving a gap in the knowledge and skills pool of the maternity section.

There is a need for clinical leaders, including trained neonatal nurses and paediatricians. Owing to staff shortage and the inability of staff to attend training off site, neonatal resuscitation training was given on site. The training consisted of an adjusted Helping Babies Breathe programme and focused on bag mask ventilation and cardiac compressions.^26,27,28^ Staff learned how to do single rescuer neonatal resuscitation, as they should be able to perform this on their own until assistance arrives. Available staff should be allocated efficiently and effectively and should be placed according to interest and skill.

Neonates often had to be transported to or from the hospital to a tertiary hospital. There was a slight improvement regarding the transport of critically ill patients from the NICU when private ambulance services were used, but this improvement was not sustained. In general, there was a slight improvement in response time, but a problem remained regarding ambulances that arrived unequipped and with emergency medical staff unable to transport critically ill neonates, in spite of explaining to the emergency call centre the condition of the neonates and the equipment and expertise needed for transport. There appeared to be a persisting lack of communication between the dispatchers and the dispatch team which was beyond the control of the hospital’s management and staff. Meetings between the hospital’s management and the emergency medical services were continuing as a high priority, with the focus on the improvement of transport of critically ill neonates, as this has a direct influence on the NMRs and MDG4.

After the implementation of the strategy addressing protocols, respondents indicated that there had been improvement regarding neonatal resuscitation protocols, but the implementation was not the same in all the units. All staff received laminated neonatal resuscitation algorithms that were displayed on the walls in the resuscitation area, and protocols regarding neonatal care were addressed according to the requirements of the respective units.

The overall NMR in the maternity section of the district hospital decreased from 46/1000 live births in 2013 to 42/1000 live births in 2014. However, the NMR in this hospital is still high when considering the MDG4 target set for SA for 7/1000 live births by 2015.^8,9^ However, it should be kept in mind that, as a referral hospital receiving critically ill neonates from a large catchment area, the NMR will always be relatively high in comparison to the average NMR of the country.

**FIGURE 2:**
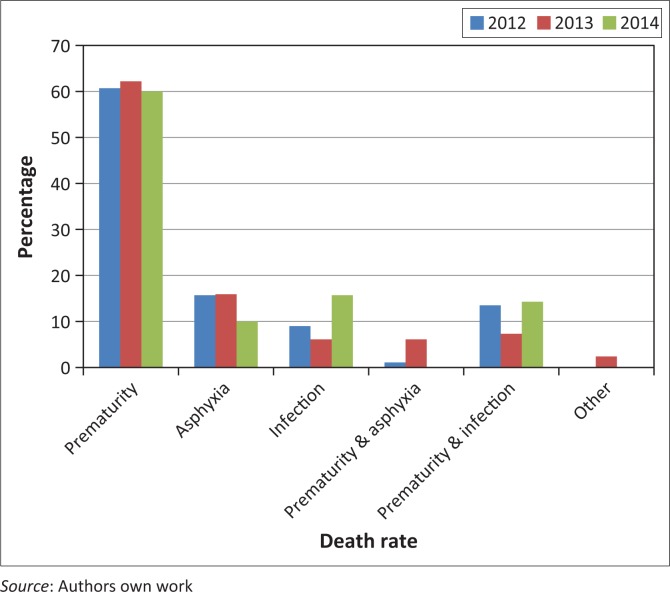
Neonatal deaths according to cause of death per year.

Prematurity remained the main cause of death. Results showed a noticeable change in the reduction of asphyxia as a cause of neonatal death, and an increase in infection as a cause of death from 2013 (illustrated in Figure 2). The reduction of asphyxia was seen as an improvement that might be related to staff training in neonatal resuscitation.

**FIGURE 3:**
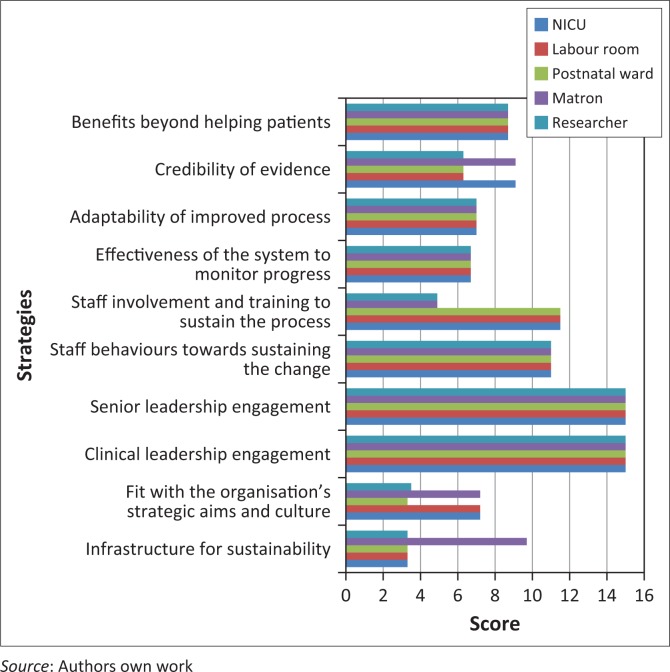
Probability of sustainability of strategies according to the Master Score System.^18^

The NHS Master Score System, which forms part of the NHS Sustainability Model and Guide,^18^ was used to determine the probability of sustainability of the strategies that were implemented. The probability for sustainability of strategies is plotted in Figure 3.

According to the NHS Master Score System, a score of 55 or higher indicates that there is a possibility for sustainability of the quality improvement initiative.^18^ The results of the last cycle indicated that all the scores added up to more than 55. Therefore, it could be concluded that the strategies implemented have the possibility to be sustained. The sustainability of the strategies is expected to contribute towards improved neonatal outcomes and decreased neonatal mortality, thereby contributing towards the attainment of SA’s drive towards the MDG4. In order to maintain sustainability the steering group should keep on motivating the staff and implementing the strategies.

## Discussion

This study created awareness within the particular district hospital, and challenges experienced related to neonatal resuscitation were identified and addressed through the implementation of strategies.

### Key findings

The sustainability of the quality improvement initiative was vital in order to create change and decrease neonatal mortality through effective neonatal resuscitation. A marked decrease in neonates admitted to the NICU died from asphyxia.

The effect of resuscitation training in facilities on the reduction of intrapartum-related neonatal deaths can be decreased by 30% and early neonatal deaths by 38%.^29^ One of the key findings ascertained that training of health care providers in neonatal resuscitation, and creating opportunities to practise on a mannequin, were successful with regards to the improvement of knowledge and skills and the confidence of staff with performing neonatal resuscitation. The hospital made some improvements towards equipment and stock availability, however there is still a need for some emergency equipment essential to neonatal resuscitation.

Staff shortages remained a problem in the context of this particular district hospital. There was a continuous potential to improve on the challenges related to the transportation of critically ill neonates to the hospital or between hospitals. Protocols regarding neonatal resuscitation were put in place. A holistic approach to quality improvement in neonatal resuscitation was followed in order to enhance the probability of sustainability.

The NHS Sustainability Model focuses on 10 essential factors related to process, staff and organisational issues. These factors play a significant role in sustaining change in health care.^18^ In this specific context this was a complex task, and the ownership and buy-in from the management and health care providers working in the maternity section were very important.

It is essential that the quality improvement initiative is sustainable and not only a short-term improvement. Staff involvement at all levels and in all stages of the quality improvement initiative is important, therefore the quality improvement initiatives should be adapted to fit the specific needs of the organisational context.^30^ Staff attitude was identified as important and played a central role in the quality improvement initiative. Allen, Greenwood and Hudson indicated that staff attitudes towards change were important with regards to the sustainability of a quality improvement initiative.^30^

Based on the results of the Master Score System, the strategies that were implemented had the potential to be sustained and could therefore contribute towards the reduction of neonatal mortality and the MDG4.

### Limitations

The study had a few limitations, one being the time constraints to implement and evaluate all the strategies, as some of the improvements would be long-term. Follow–up evaluations will continue after completion of the formal study to evaluate the sustainability of the strategies. Another limitation was the effect of staff attitude and staff involvement on change, and optimal implementation of the quality improvement initiative. The small sample size was another limitation, however all the health care providers working in the maternity section were invited to participate, and this study was context-specific.

### Recommendations

Before implementing a quality improvement imitative, there should be a steering group in place to manage the process. Ownership of all the stakeholders involved and staff attitude towards change should be placed central to a quality improvement initiative. The quality improvement initiative should be visible to all the stakeholders. Communication, teamwork and collaboration between health care providers and management are essential for quality improvement. Regarding neonatal resuscitation, it is recommended that management create neonatal resuscitation training opportunities, and address challenges in the work environment (infrastructure, culture of the organisation and staff) regarding the availability and maintenance of equipment and stock, staff shortages and protocols. It is important to address challenges related to transport of critically ill neonates to tertiary institutions. A time delay regarding transport and lack of trained emergency medical staff and equipment on ambulances are associated with increased neonatal mortality, and therefore have direct implications for the MDG4.

Recommendations for education included that all staff members working in the maternity section of the district hospital should be trained in neonatal resuscitation. Neonatal training opportunities should be created on site to ensure that all of the staff have the opportunity to attend. High frequency low impact training and practice opportunities regarding neonatal resuscitation should be created. The train-the-trainer model should be implemented to ensure continuous training opportunities and to enhance the sustainability of knowledge and skill retention. Opportunities to practise neonatal resuscitation in simulation must be created on a regular basis to contribute towards sustaining the knowledge and skills.

Training opportunities should be created regarding basic neonatal care, the recognition of early warning signs and identification of risk factors in order to prevent, or anticipate, neonatal resuscitation and to improve quality care and patient outcomes. A mentoring programme where clinical and senior leaders teach and support junior leaders can be developed and implemented which would contribute towards sustainability of the quality improvement initiative and enhance knowledge and skills. Adequate training in neonatal nursing care would contribute significantly towards the outcome and mortality rates of neonates, especially for those nurses who are working in environments and settings where there is a lack of resources and doctors and where they have to rely on their own knowledge and skills to care for the critically ill. Neonatal resuscitation should be part of the curriculums of all categories of health care staff.

Further research should be conducted regarding the role of staff attitude on the sustainability of quality improvement initiatives, and the long-term sustainability of knowledge and skill retention regarding neonatal resuscitation based on high frequency low impact training opportunities and weekly neonatal resuscitation simulation drills on a mannequin.

## Conclusion

The NMR in this district hospital was high (46/1000 live births in 2013), which might have been effected by the modifiable and avoidable causes. The development and implementation of strategies to sustain a quality improvement initiative in neonatal resuscitation was based on the theoretical framework of the NHS Sustainability Model using PRAR.^18,19^

The strategies addressed challenges with regards to neonatal resuscitation training, equipment and stock, staff attitude, staff shortages, transportation and protocols. The initiative resulted in a marked decrease in NMRs (46/1000 live births in 2013 to 42/1000 live births in 2014) as well as improvement in some of the challenges experienced related to neonatal resuscitation. This rate is still much higher than the target set for SA by the WHO for NMR to be 7/1000 by 2015.^8,9^ There is a high probability for the strategies to be sustainable according to the NHS Master Score that can effect change in practice in the long-term.^18^ If these strategies can be sustained, they could contribute to continually decreasing neonatal mortality in district hospitals in SA, thereby contributing towards the attainment of the MDG4.
